# Teacher Competencies in Health Education: Results of a Delphi Study

**DOI:** 10.1371/journal.pone.0143703

**Published:** 2015-12-02

**Authors:** Sharon Moynihan, Leena Paakkari, Raili Välimaa, Didier Jourdan, Patricia Mannix-McNamara

**Affiliations:** 1 Research Centre for Education and Professional Practice, Faculty of Education and Health Sciences, University of Limerick, Limerick, Ireland; 2 Research Center for Health Promotion, Department of Health Sciences, University of Jyväskylä, Jyväskylä, Finland; 3 Faculty of Education, ESPE Clermont-Auvergne, Blaise Pascal University, Clermont-Ferrand, France; Kyoto University, JAPAN

## Abstract

**Objective:**

The aim of this research study was to identify the core competencies for health education teachers in supporting the development of health literacy among their students.

**Method/Results:**

A three round Delphi method was employed. Experts in health education were asked to identify core competencies for school health educators. Twenty six participants from the academic field were invited to participate in the study. Twenty participants completed the first round of the Delphi, while eighteen took part in round two and fifteen participated in the final round. Data were collected using an electronic questionnaire. The first round contained an open ended question in which participants were asked to name and define all the competencies they perceived were important. Thematic analysis was undertaken on these data. A list of 36 competencies was created from this round. This list was then returned to the same participants and they were asked to rate each competency on a 7 point semantic differential scale in terms of importance. The resulting data were then analysed. For the final round, participants were presented with a list of 33 competencies and were asked to rank them again, in order of importance.

**Conclusion:**

Twelve core competencies emerged from the analysis and these competencies comprised of a mixture of knowledge, attitude and skills. The authors suggest that how these competencies are achieved and operationalised in the school context can be quite complex and multi-faceted. While the authors do not seek to generalise from the study they suggest that these competencies are an important input for all stakeholders, in order to question national and international teacher guidelines. In addition the competencies identified may provide a useful starting point for others to undertake deeper analysis of what it means to be an effective health educator in schools.

## Introduction

Recently there has been an extensive international drive to conceptualise and define core competencies for health promotion practitioners [1,2] and allied professions such as nursing [3]. Similar to the drive towards competency development as integral to the professionalisation of health promotion [4] the authors argue that consideration of competency development for the professionalisation of health education teachers is also warranted. Competencies have been defined as a combination of attributes such as knowledge, skills and attitudes which enable an individual to perform a set of tasks to an appropriate standard [1]. Competencies offer a shared language for defining what is required of the profession [5] and contribute to consolidating the discipline [1]. The potential uses of health promotion competencies include: informing advocacy for health promotion, building health promotion capacity in the workforce, developing and revising education courses, and providing a framework for credentialing in health promotion [6].

Health promotion effectiveness is dependent upon a workforce that is equipped with core, flexible and adaptable skills [2] and the recent competency framework 'Developing Competencies and Professional Standards for Health Promotion Capacity Building in Europe' (CompHP) [5] provides coherent conceptualisation of the core competencies for health promotion practitioners. The United States have a rich history of development of competencies for the health education profession [7]. They currently operate out of a model that is organized into seven areas of responsibility, further broken down into 34 competencies and then divided into 223 sub-competencies [8]. Since health promotion is delivered by different kinds of professionals in various settings, contextualising practitioner competencies is an important step in order to provide a framework for professional practice. The research in this paper is focused on moving from general health promotion practitioner competencies to more specific health education teacher competencies, relevant within the school context.

The focus on developing teacher competencies has its roots in the middle of the 20th century, when competency-based (i.e. performance-based) teacher education models became popular [9]. Some decades later, a more humanistic approach was adopted to highlight the need to focus on the process of becoming a teacher, on the teacher as a person [10] and not merely on the lists of skills that teachers require [9]. Nevertheless, “teaching professionals now face unprecedented challenges; the demands that society places on them are constantly evolving at the same time as our understanding of what makes for effective learning” [11] (page 19). This necessitates renewed understanding of the competencies teachers should have, to be able to react to these new demands. Core competencies can give coherence to the practice of teaching health [12]. Core competencies are defined as the minimum set of competencies that constitute a common baseline for all health promotion roles. They are what all practitioners are expected to be capable of doing in order to work efficiently and effectively [13].

It is important that all teachers are equipped with strong, professional competencies [14]. In order for health promotion actions to be sustainable in schools, teachers need to be capable, competent and skilled health educators. This is particularly so because they are uniquely positioned to contribute to a nation's health gain through the provision of health education to future adults [15]. Many teachers face considerable challenges in placing health education as a priority on the school agenda [16, 17]. The pressures of assessment and examinations can often eclipse the role of health education due to the finite time and space available in the school day [18]. While health education may not be the core business of schools [19], the evidence points to the fact that healthier children have better educational outcomes [20]. Thus, health education teachers need a broader range of competencies that are not only knowledge based but that also include pedagogical skills and attitudes that are conducive to the promotion of health.

The teaching of health issues in schools differs between countries, depending on political priorities as well as the organisation and goals of each particular education system [21]. In some countries health education is a subject in its own right under the responsibility of health education teachers (e.g. Finland, Ireland), while in others it is cross curricular in implementation, covered across a broad spectrum of subjects. Ideally, health education would occur within a broader framework of a Health Promoting School (HPS) [22]. The HPS framework is a specific approach that is used across countries for promoting health in the school setting [23]. It is a whole school approach aimed at enhancing the health and educational outcomes of students [23]. There are typically six components of the HPS approach; 1) healthy school policies, 2) the school's physical environment, 3) the school's social environment, 4) individual health skills and action competencies, 5) community links and 6) health services [23].

For the purposes of this paper, the concept of the health educator (or health education teacher) refers to any teacher who is involved in health matters in the school. Health promotion (HP) and health education (HE) are concepts that are often used interchangeably [24], therefore, we draw on the distinction provided by the International Union for Health Promotion and Education (IUHPE) [23]. Health promotion is defined as any activity undertaken to improve and/or protect the health of everyone in the school community including provision relating to the physical and social school environment, the curriculum, school policies and community links [23]. Health education is conceptualised as a communication activity which involves learning and teaching pertaining to knowledge, beliefs, attitudes, values, skills and competencies [23]. From this perspective health education is understood as an integral part of broader health promotion and is directed at improving health literacy [25].

"Health literacy is a key outcome measure for early child development, school curricula and lifelong learning for health and well-being that need to be promoted across the life course" ([26], p.70). Health literacy has been raised as one of the themes to be covered when developing 21^st^ century skills among pupils, in order to succeed in work and life [27, 28]. The development of health literacy is also seen as one of the main goals of modern school health programs [29]. According to Nutbeam [25] (page 357), "health literacy represents the cognitive and social skills which determine the motivation and ability of individuals to gain access to, understand and use information in ways which provide and maintain good health". It is also seen as an ability to change living conditions so as to contribute to better health for oneself and others [25, 30]. This perspective fits well in the school context, as the aim of schools includes the promotion of critical and active citizens that are able to promote not only their own health but also that of others [31].

Advancing children’s health literacy will progressively allow for greater children’s autonomy and personal empowerment [32, 33]. The process of achieving health literacy can be seen as part of an individual’s development towards improved quality of life. Schools are a targeted setting for increasing health literacy [34, 35]. Nutbeam [33] has proposed a three level hierarchy of health literacy; 1) functional health literacy, 2) interactive health literacy and 3) critical health literacy. In schools, functional health literacy involves the transmission of basic information on health topics such as hygiene, nutrition, drugs, relationships etc. [36]. Interactive health literacy involves opportunities to develop specific personal skills that enable pupils to take care of their own health and to seek health-related information. Critical health literacy is concerned with providing learning opportunities in classroom and community situations to develop critical thinking skills, and to address social inequities, determinants of health and ways of affecting change [36, 37]. Thus, the development of health literacy in schools can be seen as an outcome of health education [33] and it may take place within the strategic health promotion framework of the Health Promoting School [36].

The purpose of this study was to identify teacher competencies that are important for teachers of health education in the school setting, in supporting the development of health literacy among students.

## Materials and Methods

The Delphi method was adopted for this study. This is a survey technique, using a combination of qualitative and quantitative processes that draws on the opinions of selected experts and aims to obtain group consensus on a topic. Delphi methods have been identified as appropriate where scientific knowledge of the topic under investigation is scarce [38] and is useful when face-to face data collection is impractical [39]. A multiple round survey (three rounds in this case) was employed over a period of 10 months, to identify the core competencies that health education experts deemed to be important for teachers of health education and health promotion to promote student health literacy. This is a descriptive study on the subjective meaning of twenty international health education experts on the core competencies for health educators (of whom fifteen participated in the final round). The Delphi technique has been effectively used to examine core competencies in health promotion [40] and is advocated as a method particularly suited to the investigation of health issues [41]. It draws together the collective judgment of experts on a particular topic [42] and was chosen in this instance because, while currently there is growing international interest in teacher education for health promotion in schools [19], there is little consensus with regard to the core competencies for teachers of health education. A three round process was adopted [41, 43]. The first round comprised of an open-ended questionnaire inviting experts’ views on what competencies were relevant for health education teachers. This formed the basis of the second round questionnaire that was structured in nature [43]. The final round re-evaluated the outcomes of the second round in order to achieve consensus through prioritising and ranking the competencies identified. The reporting of this Delphi has been informed by Boulkedid et al.'s [44] systematic review on using and reporting on Delphi studies, consequently, the response rates for all rounds, the method used to achieve consensus and the specific characteristics of the participants are all reported on in this paper.

### Sample

A purposeful sample which selected ‘information rich’ participants [45] (page 230) was employed to gain expert opinions on the topic [46]. Participants were selected in line with Gutierrez's recommendation [47] that the group is knowledgeable, can provide valuable input in the process and are interested and dedicated in the field of study/practice. The aim also was to select participants who could provide various perspectives and provide a variation in responses about all the possible competencies deemed important for health education teachers in schools [45]. Hence, the reason we included participants from various countries (with differing health education practices in schools) as well as a range of related experience in the field. This was important in the first round when the purpose was to elicit as many possible competencies deemed important for health education teachers in schools.

The Delphi was performed in English and any potential language difficulties were offset by the fact that all experts were proficient in English. All participants had specific expertise in health education in schools and are also considered experts in the broader health promotion field, of which health education is an integral part. The sample were recruited based on their expertise in school health education and through their membership of the Schools for Health in Europe (SHE) Network and the International School Health Network (ISHN), which are international organisations that promote school based health education. All of those invited to participate had to have made a significant contribution to the knowledge base of health education in schools internationally. Therefore, inclusion criteria included; a) having international publications in the field of health education and/or, b) having acted as a teacher trainer or other university-level educator in school health education. It is acknowledged that the sample selection process used by the authors was subjective.

Sample sizes vary from three to several hundred participants in published Delphi studies [48].The number of experts working in this area comprises a relatively small sample and similar health promotion Delphi studies were used as a guide in determining how many participants were needed [46, 49]. Research has outlined that a Delphi panel is usually under fifty participants [50] and the literature suggests having 10–18 participants on a Delphi panel [51]. Twenty six experts were initially invited to participate in the first round. Twenty participants accepted the invitation in the first round. Anonymity is a key component of Delphi studies and therefore, the same list of twenty six experts were e-mailed each of the three rounds of the Delphi. Table 1 presents information on the sample of participants who responded to the Delphi invitation in each of the three rounds. This includes; gender, profession, country of work and qualifications.

**Table 1:** Participant Information.

	Participants	1st Round	2nd Round	3rd Round
**Gender**	Male	4	4	3
	Female	16	14	12
	**Total**	**20**	**18**	**15**
**Profession**	Lecturer in Teacher Education	11	13	10
	Lecturer (other)	-	2	2
	Professor	-	2	2
	Health Promotion consultant	1	1	1
	Researcher	8	-	-
	**Total**	**20**	**18**	**15**
**Country of work**	Australia	2	2	3
	Canada	1	-	-
	Denmark	1	1	1
	Finland	6	5	4
	France	2	2	1
	Germany	1	-	-
	Ireland	2	2	1
	Norway	2	2	2
	Portugal	1	1	1
	Sweden	1	1	1
	United Kingdom	1	2	1
	**Total**	**20**	**18**	**15**
**Qualification**	Ph.D	14		
	Masters	3		
	Bachelor degrees	3		
	**Total**	**20**		

### Data collection

Data were collected using an electronic questionnaire sent to the experts via e-mail. Anonymity was critical to the process in order to facilitate the freedom of participants to express their views on the topic. Questionnaires were sent to each participant separately in order for their e-mail addresses to remain confidential and to protect the anonymity of the participants [49]. Participants did not know the content of other responses, instead their answers were sent to a central facility for collation by the researcher [41]. This lessens any pressure to conform to a group position. After each round the results were analysed by the research team and sent back in the form of another questionnaire in the next round. The participants were shown only the combined results and not the statistical analysis or detailed results [49].

### Measurement

The first round questionnaire comprised of five questions, two of which sought demographical information (gender and country) and two of which sought level of expertise and current professional employment. The final question asked ‘what do you believe are the core competencies needed for teachers of health education in schools in supporting the development of health literacy in students?’ and asked participants to provide a short description of what they meant by each competency. The questionnaire was pre-tested with three experts from three different countries; Ireland, Turkey and Finland.

For round two, a structured questionnaire was designed based on the results of the first round and sent to the same original list of invited participants. Three questions were posed at the beginning of the questionnaire requiring demographic information and then, the competencies were listed and participants were asked to rate them on a 7 point semantic differential scale in terms of importance, 1 being ‘not at all important’ and 7 being ‘very important’. In addition, an open question was included that gave participants space to add any potential competencies that on reflection, they perceived were missing from the list.

The analysis of round two yielded a revised list of competencies that formed the basis of the instrument sent in round three. The third and final round of the Delphi asked participants to chose the top 10 competencies and rank in order of importance.

### Data analysis

In order to analyse the results of the first round and to minimise redundancy by grouping similar ideas together [52], a two stage qualitative and inductive ‘thematic analysis’ was undertaken [53]. The process employed was similar to that described by Milat et al. [46]. Two authors assessed the responses independently in order to identify broad coding themes for each competency. The coding frames were discussed and agreed and a final joint thematic analysis conducted. Another author was also consulted at this stage in order to eradicate any bias. When collated to remove repetition, in the first round analysis, thirty six potential competencies were identified. After discussion about semantic similarity [38], all three researchers agreed on the thirty six potential competencies identified. An inductive analysis was deemed appropriate, allowing trends to emerge from the data without presupposing in advance what these may be [45]. This inductive analysis approach took the form of both emic and etic analysis of the data [54]. For the majority of competencies identified emic analysis was employed where the categories were defined using the terms given by the participants. However, for certain competencies, such as those specifically in the knowledge category, the label was imposed by the researchers, known as etic analysis. For this Schulman’s [55] work on knowledge categories was consulted.

The data from round two were entered into the SPSS statistical software package and analysed. The median values and frequency distribution were calculated to examine level of agreement on each competency. Competencies that were deemed to have achieved consensus as to their importance had to have been rated ≥5 by more than 50% of all respondents and to have achieved a median of ≥5. The median was used in the second round as a measure of central tendency as the data were not normally distributed and a small sample size was used for the Delphi. Often, to assess consensus in Delphi studies, frequency distributions are used [56] and the criterion of at least 51% responding to any given response category is used to determine consensus [57].

In the third round, participants were asked to choose their top ten competencies from the list and to rate them in order of importance. The points for each competency were allocated as follows: 10 points for each number 1 ranking, 9 points for each number 2 ranking, continuing to 1 point for each number 10 ranking. If a competency did not occur in the top 10, a value of 0 was given. The points for each competency were summed up and their mean values calculated (total of all experts). As the aim for this round was for participants to rank the competencies, the mean was an appropriate measure to use in order to ascertain the average points each competency achieved. Analysis of Delphi studies in the literature outlines that *ad hoc* cut-off points are often used [58]. The authors’ decision was to employ a mean of ≥2 as a cut-off for competency inclusion in line with a similar study and analysis conducted by Kokko et al. [49] and this resulted in twelve competencies achieving this mean. Further statistical testing was not employed due to the low number of participants and the nature of the study design.

### Ethics statement

Ethical approval was granted by the University of Limerick Research and Ethics Committee. The study design, information sheet and research instruments were reviewed by the committee and deemed appropriate for use. The information sheet outlined the voluntary nature of participation and that participants could withdraw at any time during the course of the study. In this way the researchers prioritised respect for participants as advocated by Lo Biondo Wood and Haber [59].

## Results

In the first round, the panel consisted of 20 health education/promotion experts, yielding a 77% response rate. One hundred and thirty eight various responses were received from participants. These were categorised accordingly and a total of 36 competencies were identified. The 36 competencies that emerged from this round can be seen in Table 2 and are wide ranging and diverse in nature.

**Table 2:** List of Competencies from Round Two.

	List of Competencies	N	Median	Frequency ≥
**1**	**Communication skills (eg. active listening, interpersonal skills and empowering ways of conducting dialogue)**	**18**	**7**	**94.5%**
**2**	**Ethical thinking skills (the ability to analyze the consequences of one's decisions and practices on others, and empathic ability)**	**18**	**7**	**94.4%**
**3**	Ability to collaborate with pupils	17	7	94.4%
**4**	**Teacher as a 'researcher' (i.e. the ability to think and reflect critically, to use various research to develop teaching and to continuously develop as a teacher)**	**18**	**7**	**88.9%**
**5**	**General pedagogical knowledge (of planning, various ways of teaching and appropriate means of assessment, and of classroom management)**	**18**	**6.5**	**88.9%**
**6**	**General content knowledge of health issues**	**18**	**6.5**	**77.8%**
**7**	**Knowledge of health education curricula**	**18**	**6**	**94.4%**
8	Knowledge in planning, implementing and assessing whole school health promoting activities	18	6	94.4%
9	Willingness to advocate for school rules and practices that promote health, safety and sustainable development in their schools	**18**	**6**	**89.0%**
**10**	**Knowledge of learners and their characteristics**	**18**	**6**	**88.9%**
**11**	**Pedagogical health content knowledge (Knowledge of and ability to use health specific pedagogical knowledge)**	**18**	**6**	**88.9%**
12	Ability to collaborate with other school personnel	17	6	88.9%
13	Ability to collaborate with families and the community	17	6	88.9%
**14**	**Knowledge of health education/promotion theories and models**	**18**	**6**	**88.8%**
15	Skilful application of general pedagogical knowledge	17	6	83.4%
**16**	**Knowledge of the determinants affecting health**	**18**	**6**	**83.3%**
**17**	**Skills in planning, implementing and assessing whole school health promoting initiatives**	**18**	**6**	**83.3%**
**18**	**Willingness to engage in whole school and community health promoting activities**	**17**	**6**	**83.3%**
19	Willingness to display and model health promoting behaviours in their classrooms and in their actions within the school context	18	6	83.3%
20	Ability to advocate for school rules and practices that promote health, safety and sustainable development in their schools	18	6	83.3%
21	Ability to identify and address student learning problems	18	6	83.3%
22	Teachers sense of self-efficacy in teaching health issues	17	6	83.3%
23	Knowledge about the operational environment of a school (Knowledge about the school as a system and how it relates to wider society)	18	6	77.7%
24	Skills in engaging in community based approaches to health promotion	18	5	83.3%
25	Ability to acquire leadership support	17	5	77.8%
26	An awareness and ability to be able to use resources affectively	16	5	77.8%
27	Knowledge in engaging in community based approaches to health promotion	18	5	72.3%
28	Knowledge of various disciplines (related to health science or education e.g. Sociology, Psychology)	18	5	72.2%
29	General knowledge of human development	18	5	72.2%
30	Teachers' Self-Knowledge	17	5	72.2%
31	Ability to advocate for health, social and other services for their students	18	5	66.6%
32	Willingness to enforce school guidelines	17	5	61.1%
33	Ability to identify and refer pupils' illnesses and problems (other than learning problems)	17	5	55.6%
34	Willingness to help students and parents manage their illnesses or problems within the school day [Removed Round 3]	17	4	38.9%
35	Knowledge about health related careers [Removed Round 3]	17	4	22.3%
36	Ability to identify and refer fellow staffs problems [Removed Round 3]	17	3	33.3%

Eighteen health education/promotion experts participated in the second round. The competencies were listed at random for participants in order not to influence results. The items presented in Table 2 below are the results from round two which include the median and frequency distribution of each competency. The competencies highlighted in bold text in Table 2 later emerged as the most important competencies in round three. Three of the competencies achieved a median of <5 and a frequency distribution of <50% and were eliminated from the third round. Therefore, the analysis of this questionnaire formed the final competency list issued to participants in the third round.

In the final round, 15 participants responded to the questionnaire invitation. Twelve competencies achieved a mean of ≥2 which was the cut off point for inclusion as the most important competencies identified by participants. The twelve most important competencies identified by participants are outlined in Table 3 below.

**Table 3:** Most important competencies (Round Three).

Most Important Competencies	Arithmetic Mean
1. Knowledge of the determinants affecting health	4.9
2. Communication skills	4
3. Teacher as a ‘researcher’	3.9
4. Pedagogical health content knowledge	3.9
5. General content knowledge of health issues	3.9
6. General pedagogical knowledge	3.7
7. Knowledge of health education/promotion theories and models	3.7
8. Skills in planning, implementing and assessing whole school health promoting initiatives	2.9
9. Knowledge of health education curricula	2.9
10. Knowledge of learners and their characteristics	2.5
11. Ethical thinking skills	2.2
12. Willingness to engage in whole school and community health promoting activities	2

## Discussion

**Fig 1:**
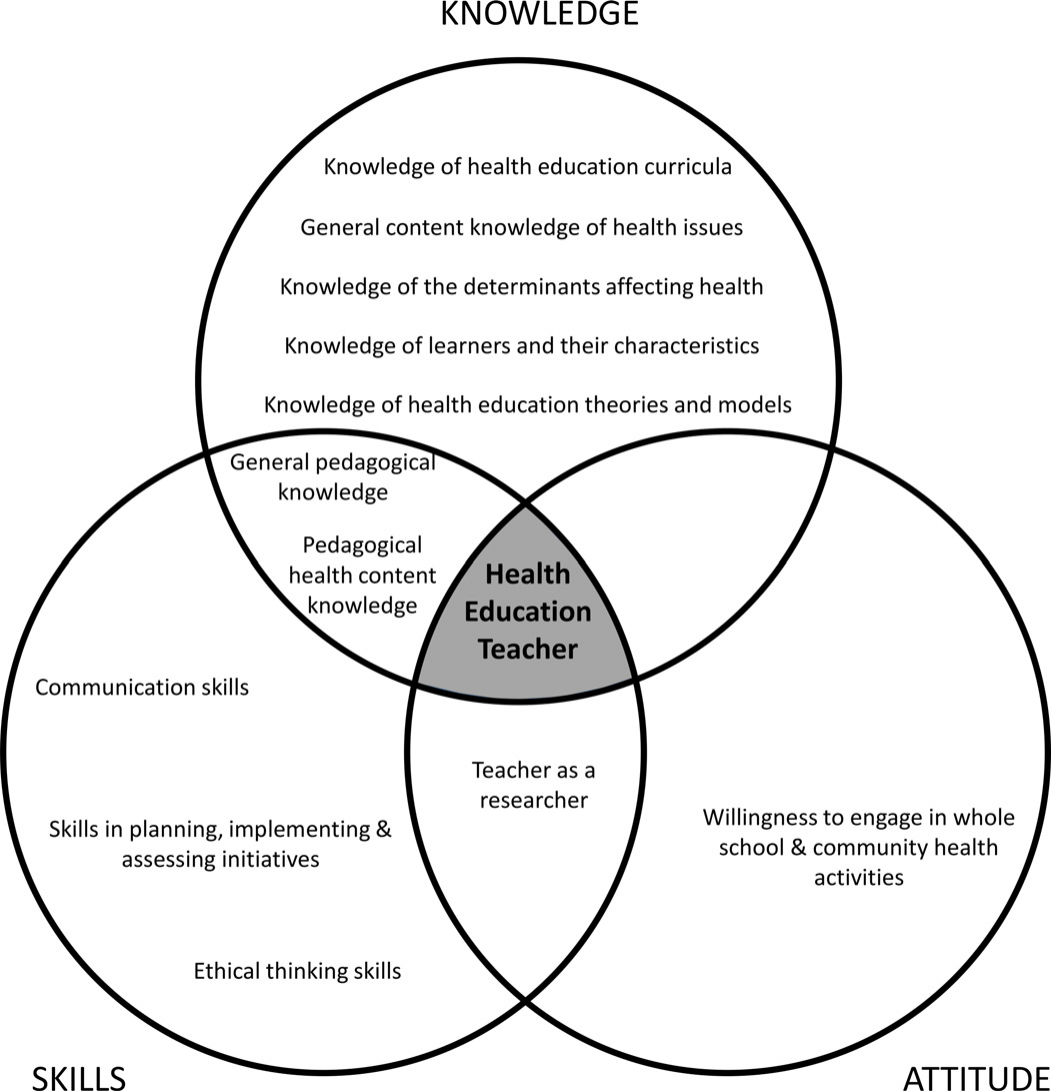
Core Competencies of Health Education Teachers.

Defining the teacher’s role in health education is complex. It lies at the intersection between the private and public domains, and is related to behavioural issues that are determined culturally [19]. Teachers have many priorities including building literacy and numeracy skills; scientific and artistic competencies; societal, historical and cultural dimensions. They are also expected to provide the means for students to succeed. With these many priorities it is not always easy for teachers to have a clear view of their contribution to the promotion of health and well-being in schools [19]. Lack of coherent conceptualisation of the competencies required for health education teachers further exacerbates this complexity. The competencies identified by the expert panel, can be categorised into three domains: knowledge, skills and attitude (see Fig 1). However, the authors suggest that how these competencies are achieved and operationalised in the school context can be quite complex, multi-faceted and at times overlapping.

### Knowledge based competencies

It is noteworthy that the knowledge domain dominated the ranking of competencies that were deemed most important by participants. This is unsurprising given that the primary focus in schools is the cognitive development of children, and the ensuing dominance of student performativity [60–62]. In recent decades the promotion of education for the knowledge economy [63] has resulted in the emphasis on the cognitive domain in schools [64, 65]. Knowledge based competencies emerged from the analysis as the most dominant, accounting for seven of the twelve competencies identified in the final round. The knowledge based competencies identified in this research resonate well with Shulman’s [55] seminal paper on knowledge and teaching. Shulman's work identifies the distinctions between content knowledge; general pedagogical knowledge, pedagogical content knowledge (PCK); curriculum knowledge; and knowledge of learners and their characteristics. In this Delphi study, content knowledge accounted for three of the competencies identified; 'content knowledge of health determinants', 'general content knowledge of health issues' and 'knowledge of health education/ promotion theories/models'. A competency specific to curriculum, 'knowledge of health education curricula' was also evident and one competency pertained to the 'knowledge of learners and their characteristics'.

The remaining knowledge based competencies comprised of pedagogical knowledge. General pedagogical knowledge can be defined as the "broad strategies and principles of classroom management and organization that appear to transcend subject matter" [55] (page 8). Pedagogical content knowledge then is more specific to the distinctive bodies of knowledge for teaching; "representing the blending of content and pedagogy into an understanding of how particular topics, problems, or issues are organized, represented and adapted to the diverse interests and abilities of learners, and presented for instruction" [55] (page 8). What should be noted here is that both PCK and general pedagogical knowledge include elements of skills, such as ‘how’ to plan, implement and assess teaching-learning situations [66], knowledge that is action-oriented and applied. It could be argued therefore that PCK and general pedagogical knowledge intertwine both knowledge and skills dimensions.

### Skills based competencies

The next domain of competencies resided in the skills sphere and included specifically 'communication skills (including active listening, interpersonal skills and empowering ways of conducting dialogue)'; 'skills in planning implementing and assessing whole school health promoting initiatives', 'ethical thinking skills' and 'teacher as a researcher'. Health communication strategies are central to the promotion of health and are in reality quite intricate. They include intrapersonal, interpersonal, organizational and community communication skills [67]. Skills in planning, implementation and assessment of whole school health promotion have also been identified in the literature as important skills for health educators [36, 68, 69]. Indeed, McKenzie et al. [67] advocate that the responsibilities of health educators’ are frequently linked to programme planning, implementation and evaluation. Ethical thinking skills delineate what is considered acceptable and unacceptable conduct regarding professional practice in health education [69]. Teaching as a profession has been recognised as ethically sensitive because teachers work with young students who may be open to influence and who may be less capable of safeguarding their rights than adults [70]. Paakkari and Välimaa [71] advocate that the ethical sensitivities become even more salient when teaching subjects that focus on pupils’ daily living, their attitudes and values, and in particular health related content. Therefore, it is appropriate that ethical thinking skills were present.

'Teacher as a researcher' is a competency that overlaps both the attitude and skills domain. Conceptualising teacher practice as having a broader professional remit that now includes research has taken hold in the past decade. Teachers are now more commonly expected to engage in research and are being described in terms of practitioner researchers [72]. Teacher research is conceptualised by Mohr et al. [73] (page 23) as "inquiry that is intentional, systematic, public, voluntary, ethical, and contextual” with the clear aim of improving teaching and contributing to better schools. Teachers are now required to have strong content knowledge with skills for reflective practice and research [74]. Indeed, the need for teachers to value research and to use it to improve practice is strongly advocated [75–77]. This resonates well for health education. The 'teacher as a researcher' will become a more prominent competency expected of teachers regardless of subject expertise. It is appropriate that it emerged as an important competency in this study, given the requirement for health education teachers to demonstrate flexibility, more experiential pedagogical expertise and engagement with the complexity of health promoting schools.

### Attitude based competency

The final competency was attitudinal in nature. The importance attributed to the competency 'willingness to engage in whole school and community health promoting activities' was unsurprising given that, health education effectiveness is dependent on teacher commitment and ability to convey enthusiasm to others [78, 79]. Teachers in schools have different approaches and varying interpretations of their role in health education [80]. This is dependent on their subject and on their personal epistemologies of education [78]. Willingness to engage in whole school and community health promotion activities is influenced by several factors not least of which are school culture and personal disposition. Teacher preparation also plays a significant role in this regard [81]. It is noteworthy that the international literature points to the need for teacher willingness to engage in the promotion of health and well-being, as central to the success of health education in schools [36, 78].

### Comparison with other competency frameworks

The range of competencies that were deemed most important by participants reflects that health education is not a simple endeavour. There were in fact specific competencies that cross three domains of teacher expertise in particular that of knowledge, skills and attitude. The competencies identified as important in this study intersect with some of those identified through the CompHP project [5]. For example, ethical values and a health promotion knowledge base underpinned the other nine competency domains in the CompHP project [5]. In this study, ethical thinking skills as well as knowledge competencies were also prioritised by participants. The knowledge categories in this study diverge from the CompHP project as they are teacher specific and include knowledge not only of health promotion theory but also educational knowledge concerning students, pedagogy and curriculum. This is to be expected given the specificity of the setting. In research conducted by Pantic and Wubbels [82] on teacher competencies, the competency domain of subject knowledge, pedagogy and curriculum was also rated by participants as very important. In the European Commission [83] framework of teacher competencies the Knowledge and Understanding domain encompasses subject matter knowledge, PCK, knowledge of students, pedagogical knowledge and curricular knowledge, all of which featured in the most important competencies list in this study for health education teachers. The prioritisation of communication skills also overlaps with the CompHP framework [5] which similarly specified the importance of skills in communication. Communication skills such as, negotiation skills and interpersonal skills are also identified as important by the European commission [83]. The importance of research skills emerged in this study and was conceptualized within the 'teacher as researcher' domain, a growing area of focus in teacher education. This is also reflected in the CompHP list of nine competency domains as 'Evaluation and Research'. In teacher competency research, the ability to critically reflect upon teachers own practice and to take responsibility for their professional development was cited as an important competency by teachers and teacher educators [82]. While 1) assessment; 2) planning and 3) implementation were defined as separate competency domains in the CompHP project, they were conceptualised by participants as part of the same competence in this study eg. 'Skills in planning, implementing and assessing whole school health promoting activities'. Without doubt the skills to plan, implement and evaluate health promoting activities are central to successful and sustainable health education in schools [84].

## Conclusion

While the authors do not seek to generalise from the data presented here, nonetheless they believe that the data provide some insight in informing others who may seek to undertake deeper analysis of what it means to be an effective health educator in schools. It is hoped that this initial study will prompt a discourse specific to health education in schools that actually engages across these three domains of knowledge, skills and attitude. Although, there are differences across countries and within countries, in terms of the implementation of health education (either health education taught as a specific subject or the health promoting school approach), these competencies provide a useful insight for working in either of these contexts. Caution is advocated here due to the omission of expertise from the United States and from Asia. Therefore, while it is not intended to generalise these competencies globally, it is hoped that they will provide an impetus for future research in the domain. In keeping with the development of competencies for health promotion more generally, the authors advocate that the development of competencies can also be used in order to strengthen health educators professional practice, by using it to inform teacher education curriculum as well as serving as a 'self-orientation tool' for teachers [82]. The authors are keenly aware of the challenges in undertaking this type of research because globally, teacher education varies significantly. School contexts also vary substantially. However, while differences exist there are similarities in the intended outcomes of health education, namely the development of health literate children and the promotion of healthy behaviour and decision making. The study reported here is a preliminary step in conceptualising teacher competencies in health education. There clearly is significantly more work to be done in order to contribute to a coherent body of knowledge in this field. It is imperative that research is conducted to listen to the voices of teachers and their students in order to ascertain what they believe are the necessary competencies for effective health education in schools.

### Limitations

The Delphi technique has been established as a valid research technique in the health arena [41]. This notwithstanding, there are certain limitations associated with this technique [85]. It has been acknowledged that using the Delphi technique can be a lengthy process due to its iterative and sequential nature [86]. For this study, there were three data collection periods and this occurred over a ten month period. It can be easy to underestimate the commitment and time involved in participating in a Delphi study and participants may find the workload too much [41], thereby affecting participation rate. Five participants in this study withdrew from the initial round to the final round. Due to this attrition, phase two and three have very small sample sizes and this is a limitation of the study. A further limitation of the study was that a non-responder questionnaire was not administered. Therefore, it was not possible to ascertain differences between participants and non-participants and reasons for drop out during the three rounds.

Powell [87] notes that the findings of any Delphi study, do not necessarily offer indisputable fact, however, they do provide stronger consensus than other types of consensus formation. For this study, the sample consisted of experts from a range of countries and potentially this could provide a different consensus than if all the participants were from the same country [88]. For this study, it was deemed appropriate to gain knowledge from various countries in order to reflect a broad perspective on the subject. The authors were not able to identify similar organisations to SHE, in the United States and Asia and they recognise this is a significant limitation in the sampling process.

The participants selected to partake in Delphi studies, are often viewed as a potential limitation [89] and it is acknowledged that the presence of a subjective selection process as well as the attrition during the study, may have led to potential selection bias. The authors reiterate that generalisations cannot be made to larger populations.

Finally, it would have been of benefit to gather data on school contexts but this was beyond the scope of the research in this instance. It is imperative that further research includes the voice of teachers in determining what they deem to be the most important competencies for health educators. It is also important to attain student perspectives on this topic. It is hoped that this data will prompt further research that may be more specific to school contexts.
